# Use of Neurodynamic or Orthopedic Tension Tests for the Diagnosis of Lumbar and Lumbosacral Radiculopathies: Study of the Diagnostic Validity

**DOI:** 10.3390/ijerph17197046

**Published:** 2020-09-26

**Authors:** Francisco Javier González Espinosa de los Monteros, Gloria Gonzalez-Medina, Elisa Maria Garrido Ardila, Juan Rodríguez Mansilla, José Paz Expósito, Petronila Oliva Ruiz

**Affiliations:** 1 Andalusian Health Service, Hospital “Puerta Universitario del Mar”, Av. Ana de Viya, 21, 11009 Cádiz, Spain; mizivaurgente@hotmail.com (F.J.G.E.d.l.M.); jose.paz.sspa@juntadeandalucia.es (J.P.E.); 2 Nursing and Physiotherapy Department, Cadiz University, Av. Ana de Viya, 52, 11009 Cadiz, Spain; petronila.oliva@uca.es; 3 Department of Medical-Surgical Therapy, Medicine Faculty, Extremadura University, 06006 Badajoz, Spain; egarridoa@unex.es (E.M.G.A.); jrodman@unex.es (J.R.M.)

**Keywords:** lumbar radiculopathy, neurodynamic tension tests, orthopedic tension tests, magnetic resonance

## Abstract

Background: Lumbar radiculopathy is a nerve root disorder whose correct diagnosis is essential. The objective of the present study was to analyze the reliability diagnostic validity of eight neurodynamic and/or orthopedic tension tests using magnetic resonance imaging as the Gold Standard. Methods: An epidemiological study of randomized consecutive cases which was observational, descriptive, transversal, double blinded and was conducted following the Standards for Reporting Diagnostic accuracy studies (STARD) declaration. The sample size was 864 participants. Internal and external validity (CI = 95%) and reliability, were calculated for all tests performed independently. The diagnostic validity of the combined and multiple tests in parallel was also calculated. Results: The analysis indicated that only two tests performed independently had external validity, but neither had reliability or precision. The Straight Leg Raise test and the Bragard test performed in a multiple parallel way showed high sensitivity (97.40%), high negative predictive value (PV− 96.64%) and external validity (Likelihood Ratio− 0.05). The combined test of the Slump test and the Dejerine’s triad had internal and external validity. Conclusions: The Straight Leg Raise test and the Bragard test performed in a multiple parallel way and the combined test of the Slump Test and the Dejerine’s triad have clinical validity to discard lumbar or lumbar-sacral radiculopathy.

## 1. Introduction

Lumbar radiculopathy is a dysfunction of the spinal nerve root that can be accompanied by pain, weakness, sensitivity and reflex disorders in the affected anatomical area [1]. These symptoms, in particular pain, cause a negative impact on the independence of the person which is a problem in today’s society [2]. This condition causes physical disability which affects the quality of life of the person and, at the same time, has an impact on the economy of the countries.

Therefore, health care professionals need valid tools to accurately and quickly diagnose this condition. This could help to minimize the possible consequences.

Diagnosis allocates a person to a group of subjects that have a disease or with reasonable certainty [3]. An incorrect diagnosis can lead to the wrong prognosis and treatment which could harm the patient [4]. In contrast, an accurate diagnosis will lead to a correct and specific treatment [5]. In clinical practice, diagnostic tests are frequently used although its validity, precision and clinical utility are unknown [3].

Lumbosacral radiculopathies’ diagnosis is generally based on the clinical history of the patient and the objective examination [6]. Therefore, these patients require a comprehensive physical and neurological assessment [7] which can avoid the unreasonable use of complementary tests [8].

Neurodynamic or orthopedic tension tests are used to examine the nerve roots of the patient. They trigger pain or other sensory symptoms that reveal the root lesion [9]. In addition, they differentiate whether the origin of the pain is neural or musculoskeletal, evaluating the mechanics and physiology of the nervous system during movement [10]. They are diagnostic tests widely used in clinical practice. Therefore, determining their validity may be the necessary basis to justify the performance of high-cost tests, such as magnetic resonance imaging, which is currently the most valid procedure for the diagnosis of lumbar radiculopathies [11]. The few studies found in the literature reveal that neurodynamic or orthopedic stress tests help the differential diagnosis of lumbar nerve root compression [12]. However, there are few studies that analyze the diagnostic validity of the tests [13] performed independently or combined. Therefore, we can affirm that there is little scientific evidence on the accuracy [14] and diagnostic precision of these tests [13,15].

In this context, we developed this research, with the aim of estimating the diagnostic validity of the following orthopedic stress tests and/or neurodynamic tests (performed individually, in combination and in parallel): Straight Leg Raise Test (SLR) or Leg Elevation Test Extended [14,16], Bragard test (B) [17], Fajersztajn test (F) [18,19], Sicard test (S) [19], Passive Neck Flexion test (PNFT) [10], Kernig (K) [20], Slump Test (S) [21] and Dejerine’s triad (DT) [19]. As well as the efficacy of these tests when they are carried out in combination or in parallel, magnetic resonance imaging was used as the Gold Standard and for comparing the results; a Magnetic Resonance Imaging (MRI) scan was used as the Gold Standard and the results were compared.

## 2. Materials and Methods

This was an epidemiological study of randomized consecutive cases which was observational, descriptive, transversal and double blinded. The research was conducted following the STARD (Standards for Reporting Diagnostic accuracy studies) guidelines for reporting diagnostic accuracy studies [22,23]. The ClinicalTrials.gov Study Identifier of the study is NCT04485572.

### 2.1. Participants

**Figure 1:**
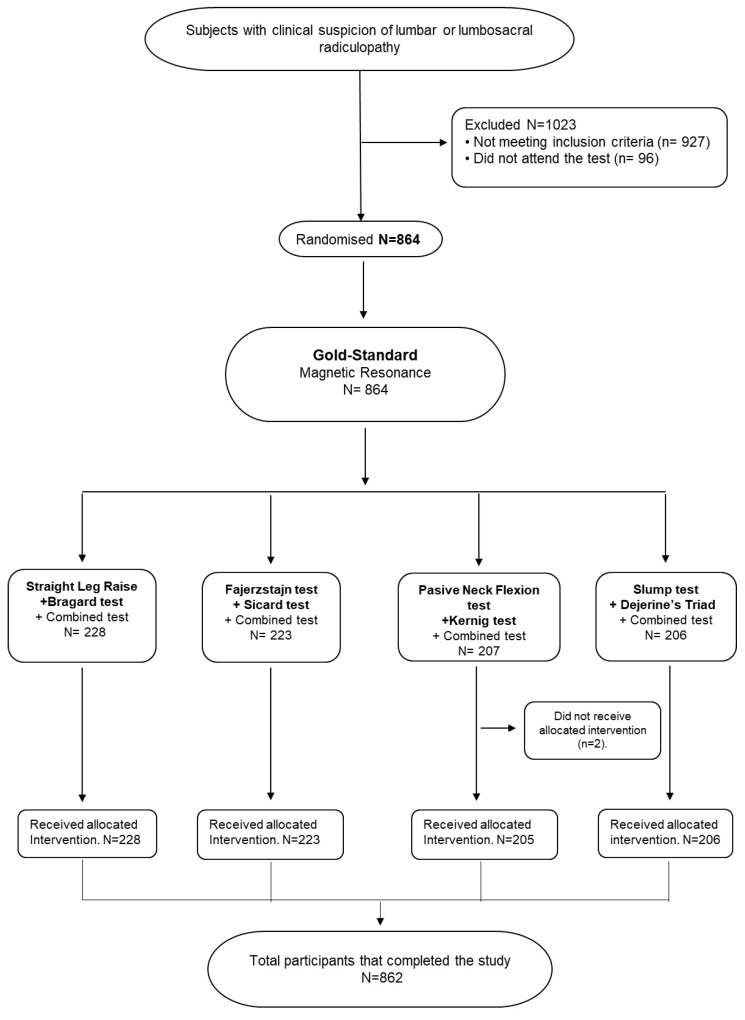
Target population.

The target population (Figure 1) included all the patients referred to the Radiology Department of the “Puerta del Mar University Hospital” in Cádiz (Spain) to undertake an MRI scan of the lumbar or lumbosacral spine (1887 subjects of which 1023 were excluded). The following inclusion criteria were established: clinical suspicion of lumbar or lumbosacral radiculopathy. The exclusion criteria were: ages under 18 or over 70 years old, healthy subjects or with a radiculopathy already diagnosed, subjects with diabetes, alcoholism, HIV+, herpes zoster infection, cancer, multiple sclerosis, hereditary neuropathy, lumbar surgery, persons with pacemaker or stent, known pregnancy and persons that refused to participate in the study or undergo the MRI scan.

The study was approved by the Bioethical Research Commission of the Puerta del Mar and Bahia de Cádiz District–La Janda University Hospital in Spain (Registration number 30/14). All the ethical considerations and requirements of human clinical research mentioned in the Helsinki declaration [24] and the Data Protection Law [25] were met.

### 2.2. Recruitment Process

Participant selection was based on the initial symptoms of lumbar or lumbosacral radiculopathy. To avoid selection bias, all the subjects referred to the Radiology Department of the “Puerta del Mar University Hospital” in Cádiz (Spain) that met the inclusion criteria were recruited consecutively and randomly.

The sample size was 864. Dividing the participants in 4 different groups was considered appropriate due to the great number of diagnostic tests that were assessed. Three diagnostic tests were performed in each group. This decision was justified and motivated by two main reasons. Firstly, neurodynamic or orthopedic tension tests trigger pain and symptoms and repeating this 12 times was not considered ethical. Secondly, systematic repetition of the tests could lead to the loss of the subjective ability of the patient to perceive changes of the symptoms.

### 2.3. Data Collection

Data collection was carried out in the Radiology Department of the “Puerta del Mar University Hospital” in Cádiz (Spain). This department belongs to the Diagnostic Imaging Clinical Management Unit of the hospital.

All the patients included in this study were subjected (after delivery of the information sheet and the signing of the informed consent) both to neurodynamic tests and to magnetic resonance imaging (Gold Standard), thereby avoiding bias sequence or diagnostic verification [3].

### 2.4. Testing Procedure

The test used as Gold Standard was MRI scan which is nowadays the test of choice to diagnose radicular pain [26]. A 1.5 Teslas Siemens MRI scan (Siemens, Erlangen, Germany) was used. The MR imaging was performed with a 4-sequence protocol performed in two planes: 1st sequence: Sagital Turbo SE-T1: TR 652, TE 14, FOV (Field-of-view) 280 mm reading, FOV phase 75%, 4 mm slice thickness, 11 slices, 4:13 min 4 h 13 min assessment time. This is the sequence of choice for morphological studies. Second sequence: Sagital Turbo SE-T2: TR 3500, TE 128, FOV 280 mm reading, FOV phase 100%, 4 mm slice thickness, 11 slices, 3:56 min assessment time. This setting is indicated to increase contrast of the spinal canal with discs and nervous structures, to visualize the connus medullaris and discs alterations and to assess spinal canal stenosis. Third sequence: Axial Turbo SE-T1: TR 438, TE 14, FOV 200 mm reading, FOV phase 5%, 4 mm slice thickness, 5 slices, 3 min and 48 s assessment time. This sequence is used for morphological studies of the medullar canal and the vertebral foramen. Fourth sequence: Axial Turbo SE-T2: TR 3970, TE 130, FOV l200 mm reading, FOV phase 5%, 4 mm slice thickness, 5 slices, 3 min and 16 s assessment time. This is a sequence which is oriented towards the study of injuries and stenosis of the spinal canal.

The images of the Gold Standard were interpreted by radiologists specialized in the diagnosis of lumbar and lumbosacral magnetic resonance images. The radiologists were staff of the musculoskeletal unit of the hospital. Two of them collaborated in the study regularly and four of them collaborated discontinuously. All of them were independent to the performance of diagnostic tests (index tests) which ensure avoidance of incorporation bias. They were blind to the results of the neurodynamic and orthopaedic tests to avoid the risk of revision bias [3].

The participants of each group were assessed with two independent diagnostic tests and a third test which was the combination of both. The combined test increases the ability of the assessor to trigger symptoms and signs by stressing or easing the different neuro-musculoskeletal structures [27]. The combination of the tests was based on their similarity of the techniques and their ability to be combined. All the tests were done bilaterally, starting with the sound limb. The tests are independent and dichotomous as they are not conditional on any other test and they have a positive or negative score.

The following neurodynamic and/or orthopedic tension tests were done: The Straight Leg Raise test (SLR) [14,16], the Bragard test (B) [17] and the combined tests of both (SLR+B); the Fajersztajn test (F) [18], the Sicard test (S) [19] and the combined tests of both (F+S); the Passive Neck Flexion test (PNFT) [10], the Kernig test (K) [20] and the tests combining both (PNFT+K); the Slump test (ST) [21], the Dejerine’s triad (DT) [19] and the test combining both (ST+DT). All tests were performed independently, combined and in a multiple parallel manner by a physiotherapist.

The clinical interpretation of the test was based on the changes of the patient’s responses in relation to the symptoms, range of movement and resistance found. The patients were informed about the importance of their perceptions [10]. The physiotherapist that conducted the tests was independent and blind to the results of the Gold Standard. To assure the validity of the results, the data of the reference test (Gold Standard) and the neurodynamic or orthopedic tension tests (index tests) were interpreted separately.

### 2.5. Statistical Analysis

The statistical analysis was carried out with the IBM SPSS Statistics Version 22 (IBM Corp., Armonk, NY, USA) and the EPIDAT 3.1 software version (Servizo Galego de Saúde, Galicia, Spain). A descriptive analysis of the age and gender of the participants was done. Their homogeneity was verified with an analysis of the variance (ANOVA) when the validity requirements allowed it or with the Kruskal–Wallis test when they did not.

To determine internal validity, the following statistical indicators were calculated for each test: sensitivity (Sens), specificity (Spec) and the positive and negative predictive values (PV+ and PV−): 0–10% null, 10–30% very low; 30–60% low; 60–70% low moderate; 70–80% high moderate; 80–90% high; 90–100% very high.

The external validity (general utility or clinical applicability for overall population) [28,29,30,31,32,33] was estimated through the Likelihood Ratio (LR+ and LR−). The range of values and their impact on the clinical utility are: LR+: > 10 great increase, excellent test; 5–10 moderate increase, good test; 2–5: small increase, bad test; <2: minor increase, useless test. LR−: 0.5–1 minor decrease, useless test; 0.2–0.5 small decrease, bad test; 0.1–0.2 moderate decrease, good test; <0.1 great decrease, excellent test [3,34,35,36]. The confidence interval for all of them was 95%. The indicators of diagnostic validity were also calculated for the test performed in the multiple parallel manner.

Moreover, in order to estimate the reliability or accuracy of the diagnostic tests, the parallel method of the same test was used. This was calculated with the Kappa index (K). The range of values were: poor, <0.20; weak, between 0.21 and 0.40; moderate, between 0.41 and 0.60; good, between 0.61 and 0.80; very good, between 0.80 and 1 [3,26,27].

## 3. Results

### Participants

The study was conducted from July 2014 until August 2016. The information related to the participants, allocated groups and tests that were performed can be seen in Table 1.

The results of the nerve root affected by group and gender are shown in Table 2.

Table 3 shows the results of the statistical indicators for all the diagnostic tests performed independently, combined and in a multiple parallel manner.

The tests assessed in this study showed no external validity (have no external applicability to this study) when they were performed independently. The SLR test and the Bragard test performed independently and in the combined test revealed internal validity. They had a high sensitivity (SLR 83.33%, B 84.38% and combined test 83.38%) and a high PV− (SLR 85.96%, B 86.61% and combined test 86.49%). However, due to the close relation to the prevalence of the condition, these results cannot be extrapolated to other populations. Therefore, the Likelihood Ratio (LR+, LR−) was calculated as this indicator is independent to the prevalence. The results obtained showed that the SLR and Bragard tests have no external validity or cannot be extrapolated to other populations, although both tests have a good reliability (0.974).

The SLR and Bragard tests performed in a multiple manner obtained high sensitivity (97.40%) and PV− (96.64%) which indicates that they have internal validity. Moreover, the results of the LR− (0.05) suggest that they have an excellent clinical utility to dismiss the condition.

No internal validity was found for the Fajersztajn and Sicard tests when they were performed individually, combined or in a multiple parallel manner. Among both tests, the Fajersztajn test had the best specificity (80.70%) and a very good accuracy or reliability (0.929).

The statistical analysis revealed that the Passive Neck Flexion test had a good internal validity as its specificity was very high (95.74%), its PV+ was high (89.74%) and the result of the LR+ was 7.41. However, the Kappa index (0.386) indicates that it has no diagnostic accuracy.

The Kernig test performed independently, combined with the passive neck flexion test and multiple in parallel, did not show internal or external validity.

The results also showed that the Slump test has internal validity with a high sensitivity (80.17%) and PV+ (82.30%). In contrast, no external validity was found for this test as the poor figures obtained in the Likelihood Ratio (LR+ 3.61, LR− 0.25) indicate. According to the Kappa index, this test has a very good reliability (0.841).

A very high specificity (96.67%) and a high PV+ (88.46%) suggest that the Dejerine’s Triad has internal validity. The values of the LR+ (5.95) for this test also indicate that it has external validity but the Kappa index (0.159) does not reveal reliability or diagnostic accuracy.

The summary of the reliability of the tests calculated with the Kappa index can be seen in Table 4.

From all the tests analyzed in our study, the combined test of the Slump test and the Dejerine’s triad is the only one that showed internal and external validity. Its sensitivity (93.97%) and PV− (90.91%) were very high, its PV+ was high (84.50%) and the value of the LR− (0.08) was excellent. All these results indicate that this combined test is effective in dismissing lumbar or lumbosacral radiculopathy. The multiple parallel test of the Slump test and the Dejerine’s triad obtained high sensitivity (84.10%) and PV+ (81.37%). However, it has no external validity, which means we cannot extrapolate these results to other populations, as the results of the LR+ (3.39) and LR− (0.21) suggest.

## 4. Discussion

A diagnostic test has clinical utility when it shows both validity and accuracy [37,38]. The results of this study indicate that the neurodynamic or orthopedic tension tests performed independently have no clinical utility.

The only combined test that showed diagnostic validity (internal and external) was the Slump test and the Dejerine’s triad. This suggests that the result could be extrapolated to other populations.

The Straight Leg Raise test and the Bragard test performed in a multiple parallel manner obtained an excellent diagnostic validity as it external and internal validity values indicate. This means that the tests can dismiss the lumbar or lumbosacral radiculopathies when its results are negative. No studies that could support these results were found in the literature.

Our study also revealed that the Straight Leg Raise test has internal validity when it is done independently. These results coincide with other studies that were conducted with high prevalence populations [39]. However, we do not coincide with other studies that have a similar design [40,41,42]. Regarding the safety of the test (predictive values), our results are not in accordance with the values found in other studies [43,44,45]. Regarding the lack of external validity of this test, we coincide with other studies that are available in the literature [41,42,46,47]. The authors of these studies also concluded that the Straight Leg Raise test has no clinical utility to confirm or dismiss the condition. Other authors also question the validity of this test [48,49]. Nevertheless, the accuracy and reliability that the test showed in our study was very high, being superior to the results shown in other studies [50,51,52].

As well as the Straight Leg Raise, the analysis showed that the Bragard test has internal validity but does not have external validity. We could not compare the findings related to this indicator as no studies were found in the literature to do so. The Bragard test also showed a very good reliability which was better than the results found by other authors [50].

The values obtained in the combined test of the Straight Leg Raise and the Bragard tests are similar to those obtained when the tests were performed independently. Only one study analyzing this tests was found in the research available. Nonetheless, its results were inferior to those in our study [53]. As mentioned above, the multiple parallel assessment of the Straight Leg Raise and the Bragard tests revealed excellent effectiveness to dismiss radiculopathy when the result of the test was negative. These findings were not able to be compared as no comparable studies were found published in the literature.

The Fajersztajn and Sicard tests performed independently, combined or in the multiple parallel way showed no external or internal validity. When comparing both tests, the Fajersztajn test obtained higher specificity and diagnostic reliability when it was done independently. The results related to the high specificity of this last test coincide with the results demonstrated by other authors [39,42,54,55,56,57]. However, no scientific evidence was found related to the performance of the Sicard test performed independently, combined with the Fajersztajn test or in a multiple parallel manner.

Regarding the Passive Neck Flexion test, although it has external and internal validity, its lack of diagnostic accuracy indicates that it is not clinically useful. Again, these results cannot be compared as there is no scientific evidence available in the literature. In addition, the Kernig test, the combination of the Passive Neck Flexion test and the Kernig test and their multiple parallel test showed no internal or external validity even though the Kernig test showed reliability and accuracy. This indicates that they have no clinical utility.

The analysis of the Slump test indicated that this test has internal validity which coincides with another study with a similar design to ours [58]. We are in accordance with other authors, in the high sensitivity of this test [12,44,59]. Although it showed very good reliability or accuracy, as it has no external validity, the results cannot be extrapolated to other populations. Based on these findings we consider that the Slump test has no clinical utility.

The Dejerine’s triad has internal and external validity but it did not show any reliability or accuracy. These results suggest that it has no clinical utility to diagnose lumbar or lumbosacral radiculopathy. The use of this test for the diagnosis of these conditions has not been assessed in previous studies.

The multiple parallel tests of the Slump test and the Dejerine’s triad have internal validity but no external validity. This indicates that they do not have clinical utility to diagnose the condition in other populations.

There are some limitations to this study. The tests were combined in pairs in order to perform the assessment. This decision was based on the fact that the neurodynamic or orthopedic tension tests trigger pain and symptoms and it was not considered ethical to over stimulate the patients. Combining all the tests among them could have shown better results to analyze which combination had the most clinical utility. Another limitation was the scarce studies related to the subject that were available in the literature. Although this made the comparison of our results difficult, it has been an opportunity to reveal gaps in the literature. We also consider that the statistical analysis could have been more complete if the reliability would have been calculated for the combined tests of all groups. For future studies, we would recommend an assessment of the accuracy of the combined test of the Slump test and the Dejerine triad as it showed to be the test with the highest diagnostic validity (internal and external).

## 5. Conclusions

Out of all the tests assessed in this study, only the combined test of Slump test and the Dejerine’s triad and the Straight Leg Raise and Bragard test performed in the multiple parallel way had diagnostic validity (internal and external). Therefore, both tests can be considered as appropriate to diagnose lumbar or lumbosacral radiculopathy. We also recommend these tests based on their low cost and the simplicity of the technique which makes the tests very easy and quick to perform in the clinical practice. However, an MRI scan is always recommended to confirm the diagnosis.

Based on our results, the following tests have no clinical utility when performed individually: The Passive Neck Flexion test, the Dejerine’s triad, the Straight Leg Raise test, the Bragard test, the Fajersztajn test, the Slump test, the Sicard test and the Kernig test.

Future research should be conducted to analyze the clinical utility of different combinations of the neurodynamic or orthopedic tension tests.

## Figures and Tables

**Table 1:** Data collection of the participants.

Group	Starting Date	Finishing Date	Tests Performed
SLR ^1^-B ^2^	7 July 2014	11 November 2014	Straight Leg Raise [14]
			Bragard test [17]
F ^3^-S ^4^	13 November 2014	10 July 2015	Fajersztajn test [18]
			Sicard test [19]
PNFT ^5^-K ^6^	13 July 2015	23 March 2016	Passive Neck Flexion test [10]
			Kernig test [20]
ST ^7^-DT ^8^	28 March 2016	25 August 2016	Slump test [21]
			Dejerine’s triad [19]

^1^ SLR: Straight Leg Raise test; ^2^ B: Bragard test; ^3^ F: Fajersztajn test; ^4^ S: Sicard test; ^5^ PNFT: Passive Neck Flexion test; ^6^ K: Kernig test; ^7^ ST: Slump test; ^8^ DT: Dejerine’s triad.

**Table 2:** Nerve root affected by group and gender.

Group	Gender (%)	L1 (%)	L2 (%)	L3 (%)	L4 (%)	L5 (%)	S1 (%)	S2 (%)	S3 (%)
SLR ^1^-B ^2^	Male 21.05	0	0	3.07	7.01	8.77	7.89	0	0
		5.70% Right-6.57% Left-3.94% Bilateral-10.52% Lost							
	Female 18.42	0	0.43	1.31	6.14	8.33	7.89	0	0
		9.21% Right-6.57% Left-0.87% Bilateral-7.45% Lost							
F ^3^-S ^4^	Male 27.80	0	0	4.03	8.96	12.55	8.52	0	0
		8.07% Right-11.21% Left-13% Bilateral-1.79% Lost							
	Female 19.28	0	0.44	1.34	7.17	6.72	7.62	0	0
		Right 8.07%-Left 8.96%-Bilateral 4.03%-Lost 2.24%							
PNFT ^5^-K ^6^	Male 23.90	0	0	0	6.82	12.19	9.6	0	0
		7.80% Right-12.68% Left-7.31% Bilateral-0.48% Lost							
	Female 30.24	0.48	0.97	4.39	9.26	13.17	11.70	0.48	0.48
		13.65% Right-15.60% Left-9.75% Bilateral-2.43% Lost							
ST ^7^-DT ^8^	Male 26.21	0	0	1.94	6.31	13.59	11.65	0.48	0.48
		9.70% Right-12.13% Left-12.62% Bilateral-0% Lost							
	Female 28.64	0	0	5.82	15.53	13.59	4.85	0	0
		8.73% Right-16.01% Left-14.56% Bilateral-0.48% Lost							

^1^ SLR: Straight Leg Raise test; ^2^ B: Bragard test; ^3^ F: Fajersztajn test; ^4^ S: Sicard test; ^5^ PNFT: Passive Neck Flexion test; ^6^ K: Kernig test; ^7^ ST: Slump test; ^8^ DT: Dejerine’s triad.

**Table 3:** Results of the validity indicators.

Test	Sens ^9^ (%)	Spec ^10^ (%)	PV+ ^11^ (%)	PV− ^12^ (%)	LR+ ^13^	LR− ^14^
SLR ^1^	83.33	74.24	70.18	85.96	3.24	0.22
B ^2^	84.38	73.48	69.83	86.61	3.18	0.21
Combined test SLR ^1^ + B ^2^	83.38	72.73	69.23	86.49	3.09	0.21
Multiple parallel SLR ^1^ and B ^2^	97.40	54.55	60.92	96.64	2.14	0.05
F ^3^	43.12	80.70	68.12	59.74	2.23	0.70
S ^4^	66.06	68.42	66.67	67.83	2.09	0.50
Combined test F ^3^ + S ^4^	46.79	78.07	67.11	60.54	2.13	0.68
Multiple parallel F ^3^ and S ^4^	80.69	55.21	63.27	74.94	1.80	0.35
PNFT ^5^	31.53	95.74	89.74	54.22	7.41	0.72
K ^6^	61.26	70.21	70.83	60.55	2.06	0.55
Combined test PNFT ^5^ + K ^6^	64.86	68.09	70.59	62.14	2.03	0.52
Multiple parallel PNFT ^5^ and K ^6^	73.47	67.22	72.58	68.21	2.24	0.39
ST ^7^	80.17	77.78	82.30	75.27	3.61	0.25
DT ^8^	19.83	96.67	88.46	48.33	5.95	0.83
Combined test ST ^7^ + DT ^8^	93.97	77.78	84.50	90.91	4.23	0.08
Multiple parallel ST ^7^ and DT ^8^	84.10	75.19	81.37	78.58	3.39	0.21

^1^ SLR: Straight Leg Raise test; ^2^ B: Bragard test; ^3^ F: Fajersztajn test; ^4^ S: Sicard test; ^5^ PNFT: Passive Neck Flexion test; ^6^ K: Kernig test; ^7^ ST: Slump test; ^8^ DT: Dejerine’s triad; ^9^ Sens: sensitivity; ^10^ Spec: specificity; ^11^ PV+: positive predictive value; ^12^ PV−: negative predictive value; ^13^ LR+: positive likelihood ratio; ^14^ LR−: negative likelihood ratio.

**Table 4:** Results of the reliability of the test.

Test	Cohen’s Kappa Index
SLR ^1^	0.974
B ^2^	0.974
F ^3^	0.929
S ^4^	0.674
PNFT ^5^	0.386
K ^6^	0.942
S ^7^	0.841
DT ^8^	0.159

^1^ SLR: Straight Leg Raise test; ^2^ B: Bragard test; ^3^ F: Fajersztajn test; ^4^ S: Sicard test; ^5^ PNFT: Passive Neck Flexion test; ^6^ K: Kernig test; ^7^ ST: Slump test; ^8^ DT: Dejerine’s triad.
